# Supplementary material for: Changes in the gut microbiome and fermentation products concurrent with enhanced longevity in acarbose-treated mice
Source: BMC Microbiol. 2019 Jun 13;19:130. doi: 10.1186/s12866-019-1494-7 (PMC6567620; doi:10.1186/s12866-019-1494-7)
Supplement: Supplementary file 2 — Taxonomic analysis of two dominant OTUs. (PDF 58 kb) [file 12866_2019_1494_MOESM2_ESM.pdf]

Taxonomic analysis of two dominant OTUs

The most conspicuous difference in the gut microbiota induced by ACA was a site-specific effect in two populations of bacteria both classified as members of family *Muribaculaceae* (See main-text Fig. 3). OTUs were classified based on an approximately 240 bp fragment of the 16S rRNA gene in the V4 hypervariable region. Using this fragment, we applied several lines of evidence to confirm that OTU-1 and OTU-4 are both members of the *Muribaculaceae* and that they are genetically distinct from cultured relatives. Classification of sequences using the method of Wang et al. [1] and the SILVA non-redundant database as a reference [2], identified both OTU-1 and OTU-4 as members of the family with 100% bootstrap support. While use of the RDP training set [3] Version 14 instead assigned these sequences to the family Porphyromonadaceae this is presumably because the *Muribaculaceae* are not recognized as a taxon in the RDP (previously reported by [4]), nor are alternative names for the clade ("S24-7" or "*Homeothermaceae*").

A follow-up phylogenetic analysis of representative amplicon sequences from two dominant OTUs was carried out using approximate maximum likelihood estimation implemented in the FastTree software (version 2.1.8, [5]) using the generalized time reversible model with twenty discrete rate categories (`-gtr -gamma` options). Approximate maximum likelihood phylogenetic estimation, using a selection of type strains in the order Bacteroidales, places OTU-1 and OTU-4 in a clade with representatives of the *Muribaculaceae* with >95% support for the topology of that node (see Fig. 1). While such a short sequence fragment is unlikely to perfectly recapitulate phylogeny—indeed, tree topology was generally weakly supported and was sensitive to both the choice of reference sequences and the evolutionary model used—we are nonetheless satisfied with the evidence for assignment of both OTU sequences to this clade; besides exceptions in the Porphyromonadaceae, Marinilabiliaceae, and *Bacteroides*, our phylogenetic reconstruction largely matches a recently proposed taxonomy of the Bacteroidales [6].

OTU-1 and OTU-4 represent uncultured genera. Over the analyzed sequence they have 89% and 92% identity, respectively, to *Muribaculum intestinalis* strain YL27, the first cultured representative of the *Muribaculaceae* [8]. A BLAST search against the NCBI non-redundant nucleotide collection did not find higher sequence similarity to any other cultured bacteria. Representative sequences for OTU-1 and OTU-4 share nucleotides at only 22 out of 244 positions (91%).

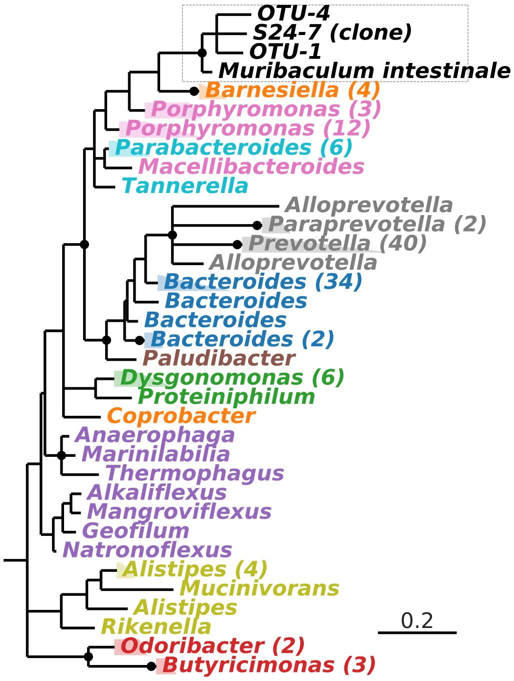

Figure 1: Phylogenetic characterization of OTU-1 and OTU-4 based on approximately 240 bp of the 16S rRNA gene V4 hypervariable region and more than 130 type strain reference sequences spanning the diversity of order Bacteroidales. Branch lengths are in units of expected substitutions per site. The tree is rooted by a Flavobacteriales out-group (not shown). Reference taxa are labeled with genus designations according to the SILVA database. When multiple representatives from the same genus have been folded together, the number of sequences is reported in parentheses. Nodes with Shimodaira-Hasegawa local support over 95% are indicated with black circles and nodes with support less than 70% have been collapsed to polytomies. The dashed box encloses taxa inferred to be within the *Muribaculaceae*. The taxon labeled 'S24-7 (clone)' (GenBank: AJ400263.1) is the environmental sequence by which the clade was originally identified, and by which it was historically named [7], while *Muribaculum intestinalis* (GenBank: KR364784.1, DSM-28989) is the first cultured representative [8]. Label colors indicate a recently proposed family membership of each reference: Prevotellaceae (gray), Barnesiellaceae (orange), Porphyromonadaceae (pink), Dysgonomonadaceae (green), Bacteroidaceae (blue), Tannerellaceae (light blue), Marnilabilaceae (purple), Marinifilaceae (red), Paludibacteraceae (brown), and Rikenellaceae (yellow) [6].

References

[1] Qiong Wang, George M. Garrity, James M. Tiedje, and James R. Cole. Naïve Bayesian classifier for rapid assignment of rRNA sequences into the new bacterial taxonomy. *Applied and Environmental Microbiology*, 73(16):5261–5267, aug 2007. URL: https://www.ncbi.nlm.nih.gov/pmc/articles/PMC1950982/, arXiv:Wang,Qiong,2007,Naive, doi:10.1128/AEM.00062-07.

[2] Pelin Yilmaz, Laura Wegener Parfrey, Pablo Yarza, Jan Gerken, Elmar Pruesse, Christian Quast, Timmy Schweer, Jörg Peplies, Wolfgang Ludwig, and Frank Oliver Glöckner. The SILVA and "all-species Living Tree Project (LTP)" taxonomic frameworks. *Nucleic Acids Research*, 42(D1):643–648, 2014. URL: https://www.ncbi.nlm.nih.gov/pmc/articles/PMC3965112/, doi:10.1093/nar/gkt1209.

[3] James R. Cole, Qiong Wang, Jordan A. Fish, Benli Chai, Donna M. McGarrell, Yanni Sun, C. Titus Brown, Andrea Porras-Alfaro, Cheryl R. Kuske, and James M. Tiedje. Ribosomal Database Project: data and tools for high throughput rRNA analysis. *Nucleic Acids Research*, 42(Database issue):D633–D642, jan 2014. URL: https://www.ncbi.nlm.nih.gov/pmc/articles/PMC3965039/, doi:10.1093/nar/gkt1244.

[4] Thomas Clavel, Ilias Lagkouvardos, Michael Blaut, and Bärbel Stecher. The mouse gut microbiome revisited: From complex diversity to model ecosystems. *International Journal of Medical Microbiology*, 306(5):316–327, 2016. doi:10.1016/j.ijmm.2016.03.002.

[5] Morgan N. Price, Paramvir S. Dehal, and Adam P. Arkin. FastTree 2 - approximately maximum - likelihood trees for large alignments. *PloS one*, 5(3):e9490, jan 2010. doi:10.1371/journal.pone.0009490.

[6] Kate L. Ormerod, David L. A. Wood, Nancy Lachner, Shaan L. Gellatly, Joshua N. Daly, Jeremy D. Parsons, Cristiana G. O. Dal'Molin, Robin W. Palfreyman, Lars K. Nielsen, Matthew A. Cooper, Mark Morrison, Philip M. Hansbro, and Philip Hugenholtz. Genomic characterization of the uncultured Bacteroidales family S24-7 inhabiting the guts of homeothermic animals. *Microbiome*, 4(1):36, 2016. URL: http://microbiomejournal.biomedcentral.com/articles/10.1186/s40168-016-0181-2, doi:10.1186/s40168-016-0181-2.

[7] Nita H. Salzman, Hendrik de Jong, Yvonne Paterson, Hermie J. M. Harmsen, Gjalt W. Welling, and Nicolaas A. Bos. Analysis of 16S libraries of mouse gastrointestinal microflora reveals a large new group of mouse intestinal bacteria. *Microbiology*, 148(11):3651–3660, 2002. URL: http://mic.microbiologyresearch.org/content/journal/micro/10.1099/00221287-148-11-3651, doi:10.1099/00221287-148-11-3651.

[8] Ilias Lagkouvardos, Rüdiger Pukall, Birte Abt, Bärbel U. Foesel, Jan P. Meier-Kolthoff, Neeraj Kumar, Anne Bresciani, Inés Martínez, Sarah Just, Caroline Ziegler, Sandrine Brugiroux, Debora Garzetti, Mareike Wenning, Thi P. N. Bui, Jun Wang, Floor Hugenholtz, Caroline M. Plugge, Daniel A. Peterson, Mathias W. Hornef, John F. Baines, Hauke Smidt, Jens Walter, Karsten Kristiansen, Henrik B. Nielsen, Dirk Haller, Jörg Overmann, Bärbel Stecher, and Thomas Clavel. The Mouse Intestinal Bacterial Collection (miBC) provides host-specific insight into cultured diversity and functional potential of the gut microbiota. *Nature Microbiology*, 1(August):16131, 2016. URL: http://www.nature.com/articles/nmicrobiol2016131, doi:10.1038/nmicrobiol.2016.131.
