# Supplementary figures and images for: Changes in the gut microbiome and fermentation products concurrent with enhanced longevity in acarbose-treated mice
Source: BMC Microbiol. 2019 Jun 13;19:130. doi: 10.1186/s12866-019-1494-7 (PMC6567620; doi:10.1186/s12866-019-1494-7)

**A**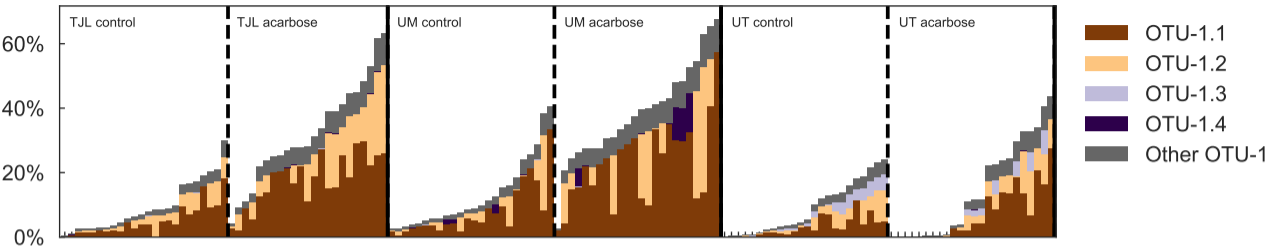**B**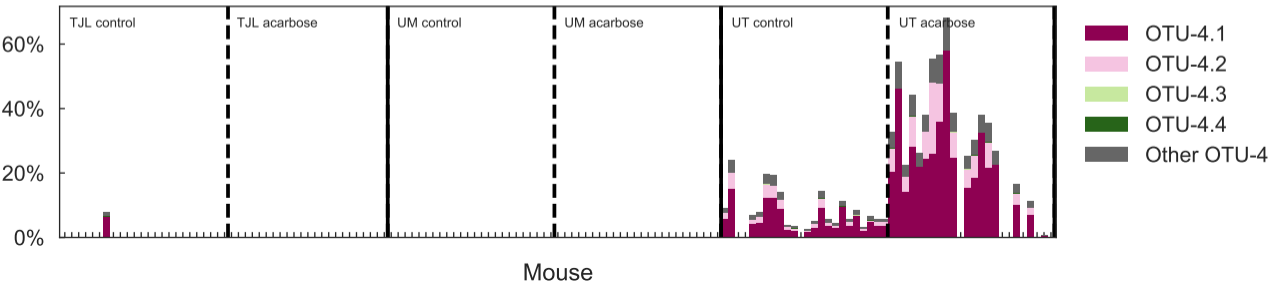

Supplement: Supplementary file 3 — Relative abundance across samples of the most common unique sequences clustered into (A) OTU-1 and (B) OTU-4. Colors are assigned to the top four most common unique sequences within each OTU and all remaining sequences from that OTU are assigned the color gray. Stacked bars in each position represent individual mice sampled for this study, and reflect the relative abundance of unique sequences in that sample. Mice are sorted by sites and then treatments, and finally by the total abundance of OTU-1. (PDF 49 kb) [file 12866_2019_1494_MOESM3_ESM.pdf]
