# Supplementary material for: Changes in the gut microbiome and fermentation products concurrent with enhanced longevity in acarbose-treated mice
Source: BMC Microbiol. 2019 Jun 13;19:130. doi: 10.1186/s12866-019-1494-7 (PMC6567620; doi:10.1186/s12866-019-1494-7)
Supplement: Supplementary file 4 — Expanded survival analysis. (PDF 44 kb) [file 12866_2019_1494_MOESM4_ESM.pdf]

Expanded survival analysis

Proportional hazards regression was used in this study to demonstrate an association between SCFA concentrations in feces and the longevity of mice. Given the limited number of samples for which matched chemical and survival data are available, statistical testing of associations with SCFAs were carried out in the pooled dataset. Therefore, to account for known effects of treatment, sex, and site, the primary null model used in this study includes terms for all main, two, and three-way interactions of the design covariates: treatment, sex, and study site. A priori, a number of these terms were expected to be non-zero, based on ITP findings from previous cohort years [1, 2]. Indeed, when survival data from all of control and ACA treated mice in this cohort were analyzed together, effects were detected that recapitulated these expectations, including: increased longevity of females, increased longevity with ACA treatment, and increased longevity of control males at UM (see Tbl. 1).

Table 1: Fitted coefficients for experimental design covariates in the full ITP cohort data.

Term	log(HR)	Std. Error	P
ACA	-0.530	0.170	0.002
Female	-0.266	0.143	0.063
TJL	-0.024	0.144	0.865
UM	-0.598	0.155	0.000
ACA:Female	0.230	0.245	0.349
ACA:TJL	-0.225	0.242	0.353
ACA:UM	0.003	0.254	0.992
Female:TJL	-0.017	0.204	0.934
Female:UM	0.580	0.213	0.006
ACA:Female:TJL	0.351	0.349	0.313
ACA:Female:UM	-0.054	0.361	0.881

Interestingly, some—though not all—of these effects were still evident when analyzing the much smaller data set of mice from which we collected fecal samples.

Table 2: Fitted coefficients for experimental design covariates in the sampled mice.

Term	log(HR)	Std. Error	P
ACA	-0.770	0.426	0.071
female	-0.239	0.421	0.570
UM	-0.931	0.439	0.034
ACA:female	0.398	0.589	0.498
ACA:UM	0.146	0.608	0.810
female:UM	0.807	0.597	0.176
ACA:female:UM	0.187	0.845	0.825

This increased our confidence that, despite the age of the mice at the time of entry and the limited sample

size, the associations with SCFA concentrations reflect patterns that could be seen in the full cohort.

As described in the main results, adding terms for the concentrations of three SCFAs improved the fit of the model (see Tbl. 3).

Table 3: Fitted coefficients for experimental design covariates and SCFAs in the sampled mice.

Term	log(HR)	Std. Error	P
propionate	-0.292	0.116	0.012
butyrate	-0.119	0.055	0.030
acetate	0.062	0.030	0.042
ACA	-0.124	0.488	0.799
female	-0.476	0.433	0.272
UM	-1.232	0.484	0.011
ACA:female	0.095	0.597	0.874
ACA:UM	0.241	0.654	0.713
female:UM	0.913	0.614	0.137
ACA:female:UM	0.111	0.850	0.896

Interestingly, the positive association between ACA and longevity is distinctly weakened when SCFAs are included (estimated coefficient went from -0.770 without SCFAs to -0.124 with). Although we do not carry out a formal path analysis, this is consistent with the causal effects of ACA on longevity being mediated by SCFA concentrations.

Survival analysis is potentially sensitivity to deviations from the assumptions of the Cox family of models [3]. We therefore tested proportionality and linearity assumptions relevant to our main findings. The Cox proportional hazards model assumes that the hazard associated with each covariate is proportional across the full set of ages at which mice are being tracked—e.g. that the proportional decrease in the risk of death for mice treated with ACA is equal from the first entry time to the last exit. We checked this assumption using a test of the correlation between the scaled Schoenfeld residuals and Kaplan-Meier transformed survival times (implemented as the `cox.zph` function in the survival package for R [4]) and found no evidence for deviations for any of the design parameters nor for the included SCFAs.

Table 4: Tests of non-proportionality for experimental design covariates and SCFAs in the sampled mice.

Term	Correlation	P
propionate	-0.127	0.155
butyrate	0.039	0.633
acetate	0.012	0.885
ACA	0.047	0.630
female	0.065	0.518
UM	-0.036	0.701
ACA:female	-0.090	0.401
ACA:UM	0.044	0.643
female:UM	-0.028	0.778
ACA:female:UM	0.037	0.726
GLOBAL	—	0.922

Similarly, visual inspection of residual plots did not provide any evidence of deviations from linearity assumptions inherent to the model (Fig. 1).

While regression coefficients can be directly interpreted as a proportional increase or decrease in hazard of death at all time points, in the general case, this does not equate to a proportional change in expected

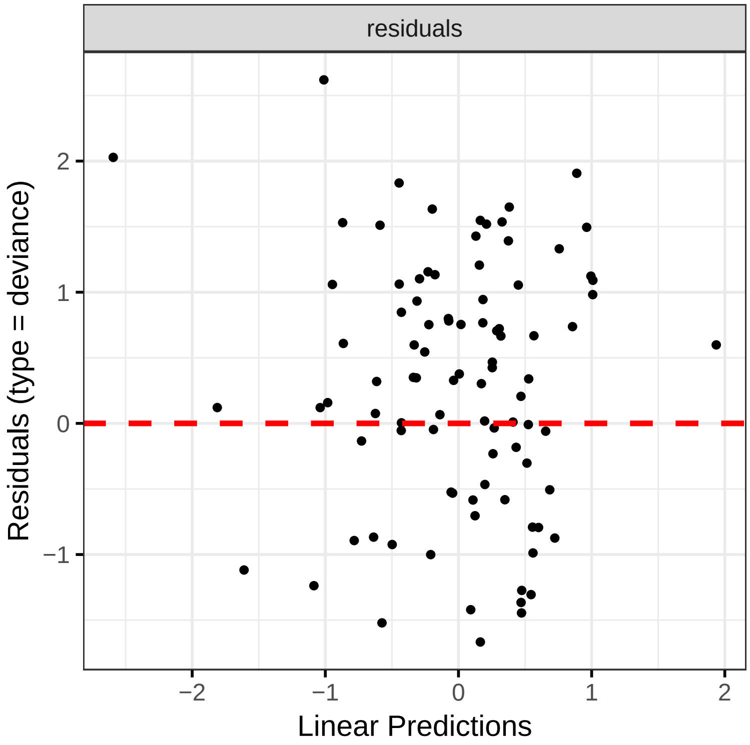

Figure 1: Plot of deviance residuals versus predicted log-hazard in a model of mouse survival that includes all design parameters (site, sex, and treatment) as well as the three SCFAs: propionate, butyrate, and acetate.

survival time. It can therefore be challenging to understand the magnitude of the survival effect on expected lifespan. To provide a more intuitive demonstration of the size of the effect, we compared predicted survival curves for ACA treated, male mice at UM, with different SCFA concentrations characteristic of two existing individuals, based on a hypothetical scenario in which mice were alive at 830 days of age (i.e. a conditional expected survival curve; see Fig. 2). These simulated results demonstrate the strength of the association between SCFAs and survival over observed differences in concentrations.

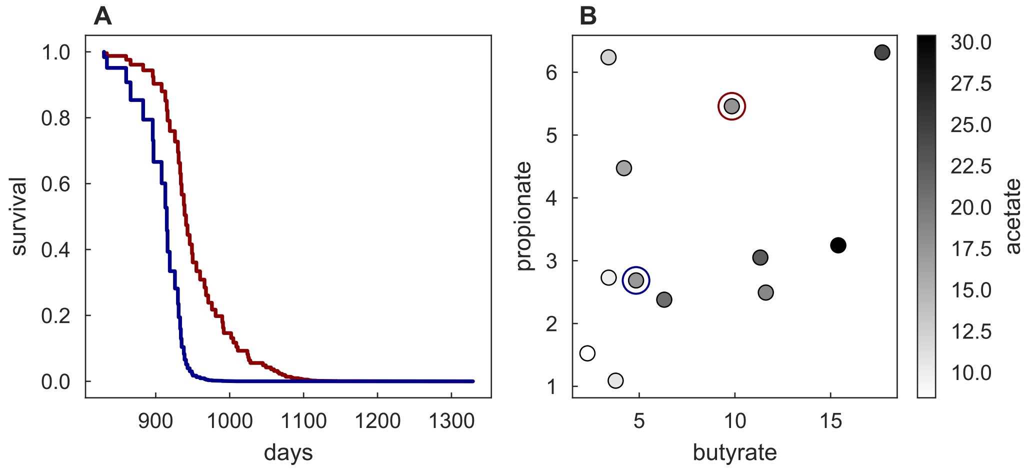

Figure 2: Predicted survival of mice exhibiting realistic variation in SCFA concentrations. (A) Expected survival curves for male, ACA-treated mice at UM, conditional on being alive at 830 days of age, and (B) SCFA concentrations for that same set of mice. Two representative butyrate, propionate, and acetate concentrations were chosen to match the measured concentrations for a high (red) and low (blue) butyrate/propionate individual, both having similar acetate concentrations.

References

[1] David E. Harrison, Randy Strong, David B. Allison, Bruce N. Ames, Clinton M. Astle, Hani Atamna, Elizabeth Fernandez, Kevin Flurkey, Martin A. Javors, Nancy L. Nadon, James F. Nelson, Scott Pletcher, James W. Simpkins, Daniel L. Smith, J. Erby Wilkinson, and Richard A. Miller. Acarbose, 17-α-estradiol, and nordihydroguaiaretic acid extend mouse lifespan preferentially in males. *Aging Cell*, 13(2):273–282, apr 2014. URL: http://doi.wiley.com/10.1111/acel.12170, `doi:10.1111/acel.12170`.

[2] Randy Strong, Richard A. Miller, Adam Antebi, Clinton M. Astle, Molly Bogue, Martin S. Denzel, Elizabeth Fernandez, Kevin Flurkey, Karyn L. Hamilton, Dudley W. Lamming, Martin A. Javors, João Pedro de Magalhães, Paul Anthony Martinez, Joe M. McCord, Benjamin F. Miller, Michael Müller, James F. Nelson, Juliet Ndukum, G. Ed Rainger, Arlan Richardson, David M. Sabatini, Adam B. Salmon, James W. Simpkins, Wilma T. Steegenga, Nancy L. Nadon, and David E. Harrison. Longer lifespan in male mice treated with a weakly estrogenic agonist, an antioxidant, an α-glucosidase inhibitor or a Nrf2-inducer. *Aging Cell*, 15(5):872–884, 2016. `doi:10.1111/acel.12496`.

[3] Patricia M. Grambsch and Terry M. Therneau. Proportional hazards tests and diagnostics based on weighted residuals. *Biometrika*, 81(3):515–526, 1994. URL: https://academic.oup.com/biomet/article-lookup/doi/10.1093/biomet/81.3.515, `doi:10.1093/biomet/81.3.515`.

[4] Terry M. Therneau. A Package for Survival Analysis in S, 2015. URL: https://cran.r-project.org/package=survival.
